# Long-term results following electrical stimulation of the peroneal nerve using the ActiGait® system in 33 patients with central drop foot

**DOI:** 10.1515/iss-2019-1003

**Published:** 2021-01-08

**Authors:** Daniel Martin, Andrei Patriciu, Anne-Kathrin Schulz, Gabriele Schackert

**Affiliations:** Department of Neurosurgery, Technische Universität Dresden, Dresden, Germany; nstim Services GmbH Vienna, Vienna, Austria

**Keywords:** ActiGait® system, drop foot, multiple sclerosis, neuromodulation, stroke

## Abstract

**Objective:**

Direct electrical stimulation of the peroneal nerve, using the implantable ActiGait® system, enables a therapy of the centrally caused drop foot, to improve the gait of the patients. In this paper, we present long-term results at 36-month follow-up post implantation.

**Method:**

A total of 33 patients, 27 stroke and six multiple sclerosis (MS) patients, suffering from spastic drop foot were implanted in our center and assessed in terms of gait endurance, speed, risk of fall, and life quality at baseline and 36 months following implantation.

**Results:**

The six min gait endurance test increased significantly from 202 ± 41 m without walking aids to 380 ± 30 m (p=0.038), while using the implant. Moreover, the time in the gait speed measured over 20 m decreased from 31.8 ± 10.2 s without to 18.5 ± 4.6 s by using the ActiGait® system (p=0.039). Similarly, gait steadiness, measured by the Timed Up and Go (TUG) test improved by 36.6%, with patients demonstrating a reduced time from 18.6 ± 5.5 to 11.2 ±  3.8 s (p=0.041) upon implant activation. Most importantly, 31 of 33 patients reported remarkable improvements of their quality of life following direct electrical nerve stimulation.

**Conclusion:**

Our findings confirm previously published efficacy data at 12 months after implantation and underline the long-lasting effect of the ActiGait® system.

## Introduction

Limitations in the gait pattern caused by drop foot compromises significantly the patients’ quality of life. Activities of daily living (ADL) as well as social life are impaired by the reduced mobility [1], [2], [3], [4], [5]. The origin of the drop foot is either central or peripheral. In case of a central origin, the upper motor neuron is affected due to neuronal damage or degeneration, which results in a spastic hemiparesis in stroke patients or monoparesis in multiple sclerosis (MS) patients [6].

The current options for the treatment of the drop foot syndrome are a foot orthosis or an electrical surface stimulator of the peroneal nerve. The development of the implantable peroneal nerve stimulation system ActiGait® opened a new treatment opportunity. Selective electrical stimulation applied to the fascicles of the peroneal nerve through a four channel cuff electrode proximal to the knee joint contracts the ankle dorsiflexor and everter muscles followed by a balanced dorsiflexion of the foot. This prospective study, performed on 27 stroke and 6 MS patients, shows long-term results at 36 months after implantation of the ActiGait®system. We previously reported elsewhere the short-term results for both patient groups [7], [8].

### Patients and methods

#### Patients’ characteristics

Twenty-seven patients with a stroke-related drop foot of at least 6-months duration and six patients with at least one year stable MS (less than three relapses) and two years of persisting spastic paresis of the leg with drop foot were offered the therapeutic option of an implantable drop foot stimulator. The mean age of stroke patients was 54 years with a range between 24 and 66 years. For the MS patients mean age was 51 with a range between 34 and 65 years. The mean time between the occurrence of drop foot and initiation of the treatment was 47 months (Table 1). Patient’s drop foot was diagnosed according to the following criteria:

1. hemiparesis persisting for at least 6 months,
2. walking ability of 10 m in less than 1 min with or without a walking aid, but without the help of another person
3. reduced speed of walking
4. positive response to surface electrical stimulation of the peroneal nerve, i.e. muscle contraction resulting in ankle dorsiflexion and the ability of achieving normal heel contact.

**Table 1:** Patients’ characteristics and individual stimulation parameters with the implantable ActiGait® drop foot stimulator.

Case no.	Age (y)	Sex	Cause of drop foot	Duration of drop foot (m)	Parameters of stimulation
1	65	M	MS	10	Channels 1 & 4 active; 1 mA; 30 Hz; optimal impulse duration 70 μs; heel switch, ipsilat
2	62	F	MS	7	Channel 1 active; 1 mA; 20 Hz; optimal impulse duration 70 μs; heel switch, ipsilat
3	47	F	MS	6	Channel 4 active; 1 mA; 20 Hz; optimal impulse duration 70 μs; heel switch, ipsilat
4	34	M	MS	15	Channel 4 active; 20 Hz; optimal impulse duration 60 μs; heel switch, ipsilat
5	48	F	MS	5	Channels 1 and 4 active; 25 Hz; optimal impulse duration 65 μs; heel switch, ipsilat
6	54	F	MS	9	Channel 4 active; 25 Hz; optimal impulse duration 70 μs; heel switch, ipsilat
7	53	M	Stroke	52	Channel 1 active; 1 mA; 30 Hz; optimal impulse duration 330 μs; heel switch ipsilat
8	62	M	Stroke	221	Channel 4 active; 1 mA; 30 Hz; optimal impulse duration 180 μs; heel switch ipsilat
9	29	F	Stroke	48	Channel 1+4 active; 1 mA; 35 Hz; optimal impulse duration 60/70 μs; heel switch ipsilat
10	58	M	Stroke	16	Channel 1 active; 1 mA; 30 Hz; optimal impulse duration 90 μs; heel switch ipsilat
11	25	M	Hem.	80	Channel 1 active; 1 mA; 30 Hz; optimal impulse duration 90 μs; heel switch ipsilat
12	59	M	Stroke	23	Channel 1 active; 1 mA; 30 Hz; optimal impulse duration 80 μs; heel switch ipsilat
13	64	F	Stroke	11	Channel 1+4 active; 1 mA; 30 Hz; optimal impulse duration 100/75 μs; heel switch ipsilat
14	36	F	Hem.	54	Channel 4 active; 1 mA; 30 Hz; optimal impulse duration 80 μs; heel switch ipsilat
15	57	F	Stroke	27	Channel 4 active; 1 mA; 30 Hz; optimal impulse duration 90 μs; heel switch ipsilat
16	36	F	Stroke	47	Channel 1active; 1 mA; 30 Hz; optimal impulse duration 330 μs; heel switch
17	24	F	Stroke	55	Channel 1+3+4 active; 1 mA; 30 Hz; optimal impulse duration 85/65/45 μs; heel switch ipsilat
18	52	F	Stroke	71	Channel 1 active; 1 mA; 20 Hz; optimal impulse duration 150 μs; heel switch ipsilat
19	58	M	Stroke	19	Channel 1+4 active; 1 mA; 30 Hz; optimal impulse duration 110/60 μs; heel switch contralat
20	49	F	Stroke	244	Channel 4 active; 1 mA; 35 Hz; optimal impulse duration 200 μs; heel switch contralat
21	54	M	Hem.	6	Channel 4 active; 1 mA; 20 Hz; optimal impulse duration 150 μs; heel switch ipsilat
22	66	F	Stroke	90	Channel 1+4 active; 1 mA; 20 Hz; optimal impulse duration 110/90 μs; heel switch ipsilat
23	54	F	Stroke	138	Channel 4 active; 1 mA; 20 Hz; optimal impulse duration 120 μs; heel switch contralat
24	64	F	Stroke	17	Channel 1+4 active; 1 mA; 20 Hz; optimal impulse duration 105 μs; heel switch ipsilat
25	43	F	Hem.	60	Channel 1+3+4 active; 1 mA; 20 Hz; optimal impulse duration 160/150 μs; heel switch ipsilat
26	50	M	Hem.	55	Channel 1 active; 1 mA; 30 Hz; optimal impulse duration 60 μs; heel switch ipsilat
27	59	M	Stroke	37	Channel 1 active; 1 mA; 30 Hz; optimal impulse duration 60 μs; heel switch ipsilat
28	54	M	Stroke	70	Channel 3 active; 1 mA; 25 Hz; optimal impulse duration 145 μs; heel switch ipsilat
29	55	M	Stroke	20	Channel 4 active; 1 mA; 30 Hz; optimal impulse duration 110 μs; heel switch contralat
30	54	F	Stroke	97	Channel 3 active; 1 mA; 25 Hz; optimal impulse duration 145 μs; heel switch ipsilat
31	48	M	Stroke	25	Channel 1+4 active; 1 mA; 30 Hz; optimal impulse duration 125/95 μs; heel switch ipsilat
32	60	M	Hem.	54	Channel 1 active; 1 mA; 25 Hz; optimal impulse duration 150 μs; heel switch ipsilat
33	55	M	Stroke	63	Channel 1 active; 1 mA; 20 Hz; optimal impulse duration 130 μs; heel switch ipsila

(MS=Multiple sclerosis; Hem = Hemorrhagic Stroke).

The gait improvement with increased foot lift was tested for each patient during the screening process after the ActiGait® implantation, using a commercially available surface stimulator (CEFAR Step II, Microstim K&T, BioNess L300, MyGait). Prior to implantation, the integrity of the peroneal nerve was assessed by means of electrophysiological testing by measuring the nerve conduction velocity (NCV) and the surface EMG. A pre-operative MRI was conducted to evaluate the peroneal nerve anatomy. The study was approved by the ethics committee of the Technical University of Dresden.

### Gait tests

Gait speed was assessed using the 20 m gait test. Patients walked 20 m at normal and maximum speed, while the time was taken. It was performed pre-operatively without walking aids, and repeated once by using an orthosis and another time by use of a surface stimulation. The same gait test setup was repeated after the implantation of the ActiGait® system after 6 weeks, and after 6, 12 and 36 months.

The endurance of the gait was assessed by the six-min Test. The distance was measured while the patients were walking for 6 min before and after the implantation with and without walking aids (orthosis, surface stimulation, ActiGait® system). Patient’s risk to stumble and fall depend on the reliability and reaction time of the system. Therefore another test “TUG” [6] was performed pre- and post-implantation. Patients were sitting on a chair and were asked to rise, walk 3 m, return and sit down. The recorded time reflects the reaction time of the system.

### Changes in the quality of life

The influence of the gait improvement on patients’ quality of life was evaluated using a questionnaire filled out pre-operatively, 6 weeks, 6, 12 and 36 months following implantation.

### The ActiGait® system

**Figure 1:**
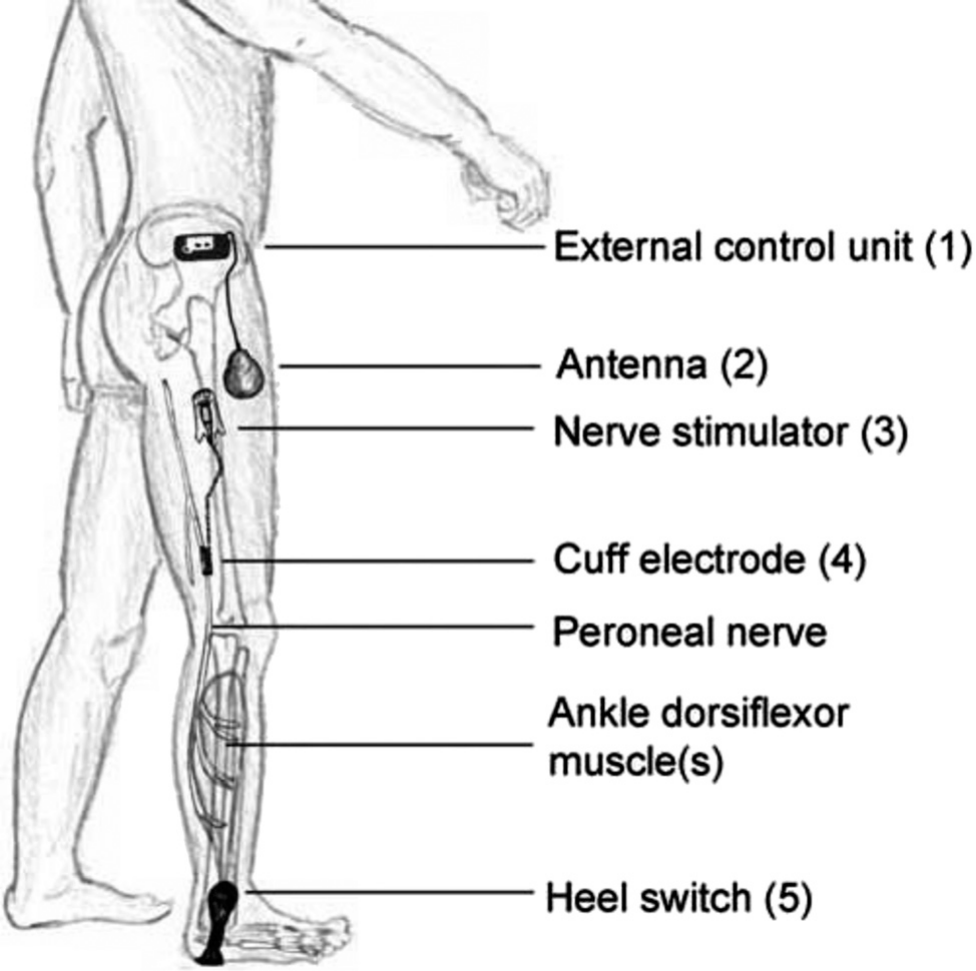
Schematic illustration of the implantable and non-implantable components of the ActiGait®-drop foot stimulator. The system consists of an external control unit (1) with a transmission coil (antenna) (2), which receives signals from the external footswitch (5) via a wireless radiofrequency signal. It enables precise activation depending on the gait cycle and allows for the adjustment of the stimulation parameters. The subcutaneously implanted stimulator (3) is connected to the 4-channel cuff (4), which is placed around the peroneal nerve. The stimulator communicates via a wireless radio frequency signal with the antenna and transmits the stimulation signals.

The peroneal nerve stimulator ActiGait® is a partly implantable system consisting of external components and an implantable stimulator (Figure 1). The latter, comprising the cuff electrode connected to the stimulator body through an electrode cable is implanted in the thigh of the patient. The cuff electrode has four stimulation channels positioned at 90° to each other allowing a 360° spatial control over nerve stimulation. This allows the optimal selection of stimulation channels during the system activation. The external control unit delivers energy and control signals over the external antenna to the implant. Stimulation is synchronized with the gait pattern being triggered by an external heel switch placed in the shoe. The therapist can select the stimulation channels to obtain a balanced foot lift and can adjust stimulation intensity and frequency using the clinical interface and computer software.

### Surgical procedure

The MRI performed prior to the implantation shows the peroneal nerve path and the level of bifurcation of the sensitive branches in the fossa poplitea region, where the cuff electrode should be placed around the nerve. The surgery is performed under general anesthesia with patients placed in a side position. Two incisions are performed to place the cuff electrode and stimulator body. The common peroneal nerve is dissected over a length of 4 cm above the tibial plateau. The stimulator body is sutured on the muscle fascia in the thigh region and the connecting electrode cable is tunneled under the skin over the biceps tendon between the two incisions. After placing and closing the cuff electrode around the motor branch of the peroneal nerve, the system is functionally tested, and finally the wound is closed. A detailed description of the procedure can be found elsewhere [9].

### Postoperative care

The system was activated four weeks after implantation, while incisions have been healed and cuff electrodes are fully encapsulated in scar tissue to avoid sliding and rotation around the peroneal nerve, and allow a stable and reliable stimulation. During activation all individual channels are tested in order to determine the foot movement and finally ensure an optimal foot lift (dorsalflexion) and balance (eversion or inversion). In addition, stimulation intensity (pulse width), ramp-up and frequency were optimized to give a balanced and sufficient foot lift. Follow-ups to optimally re-adjust stimulation were performed every 3 month during the first year and later on annually.

### Statistical analysis

Measured values are indicated with mean and standard deviation. Statistical significance was tested using the Wilcoxon rank-sum method using SPSS statistical software (ver.22 IBM). Significance value was set to p<0.05*.*

## Results

All implantations were performed according to the procedure. Activations were initialized on average 5 weeks after implantation. Optimal gait pattern was obtained using one or maximally two active channels. Most patients were programmed using channel 1 and 4. The system turned out to be easily controllable by patients and no major technical problems occurred except for cases of heel switch malfunctioning.

### Gait endurance (The six-min walking test)

The 33 patients covered in average a distance of 202 ± 41 m during the 6 min walk without walking aids. With the aid of the ActiGait® system, the covered distance increased to 380 ± 30 m (p=0.038). There was no difference in the distance by comparing measurements after 6, 12 and 36 months (p>0.05).

### The 20 m gait test

For all 33 patients, the average time required to cover 20 m in a normal gait without walking aids decreased significantly from 31.8 ± 10.2 to 18.5 ± 4.6 s after the implantation (p=0.039) and was stable over 6, 12, and 36 months.

The results of this test by using an orthosis (27.3 ± 8.3 s) or surface stimulation (23.9 ± 6.5 s) suggest similarly a positive trend with increased speed. However, this was not as pronounced as observed after the ActiGait® implantation.

### The timed up and go test

In the 36 months post-implantation follow-up, the test required 18.6 ± 4.8 s after switching off the system, which means a functionally relevant mobility impairment. Upon switching on the ActiAGait® system, the time was reduced to 11.8 ± 3.1 s (p=0.041). Notably, a similar trend was observed after 12 months post-implantation [7], [8], which underlines the stability of the system.

### Complications

Overall, we observed a few complications during our survey. In two of the 33 patients, postoperative peroneal nerve lesions were noticed, which completely recovered within 24 months following implantation. In both cases immediate re-surgery was performed to exclude a cuff electrode dislocation. One patient suffered from an electrode cable breakage. The problem was solved by replacing the implant. There occurred one case of hematoma around the cuff electrode, three cases of wound healing problems and one case of edema in the thigh region. No irreversible peroneal nerve injuries were observed.

### Patient satisfaction survey and subjective quality of life

**Figure 2:**
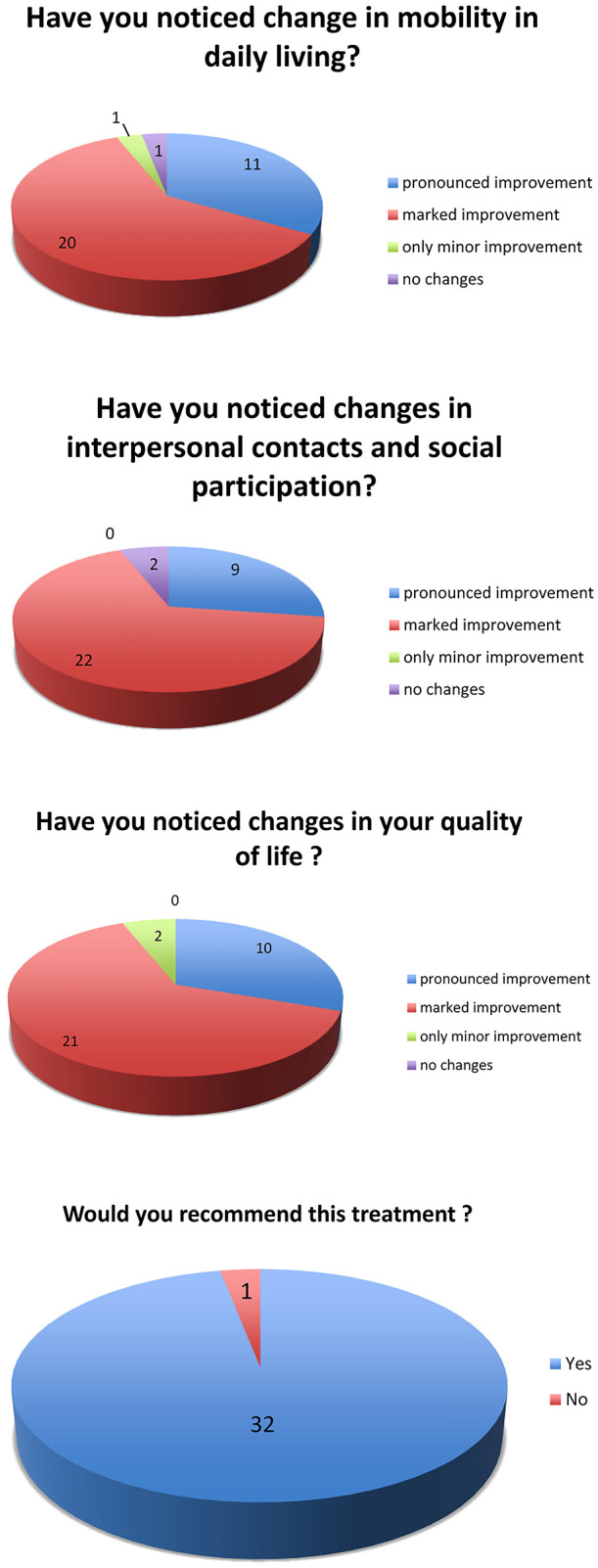
(a) Quality of life questionnaire. (b) Quality of life questionnaire. (c) Quality of life questionnaire. (d) Quality of life questionnaire.

To assess subjective changes in daily living after implantation of the ActiGait® stimulator, patients were asked to answer four questions related to QoL:

1) Did you notice any changes in mobility in the daily living? (Figure 2a),
2) Did you notice any changes in interpersonal contacts and social participation? (Figure 2b)
3) Did you notice any changes in your quality of life? (Figure 2c)
4) Would you recommend this treatment for other patients? (Figure 2d)

Thirty-one of the 33 patients reported remarkable improvements of their quality of life following direct electrical nerve stimulation (Figure 2).

## Discussion

Therapeutic options for the treatment of the centrally conditioned drop foot include the application of an orthosis around the ankle or an electrical surface stimulation of the peroneal nerve. The use of an ankle orthosis leads to stiffening of the ankle joint impeding a physiological gait pattern [9], [10], [11]. Patients are able to improve their walking compared to the time without an aid, but long-term impairment was reported following the pathologic walk pattern, which implicates an uneven load of the musculo-skeletal system.

Our study presents the 36 months long-term results of an implanted drop foot stimulator system. It is a follow-up on the previously presented results of the 27 stroke and 6 MS patients provided with an ActiGait® system.

The 36 months data show a reliably functioning system over three years, while only a few complications were observed. The complications during our survey included two cases with reversible peroneal nerve lesion, one with hematoma and three cases with wound healing problems. All patients were able to control the ActiGait® system reporting an easier use as compared to external surface drop foot stimulators tested in the patient selection phase.

Long-term results of the walking speed, measured 36 months after implantation, indicate a significant increase of 41.8% (from 31.8 to 18.5 s for 20 m normal gait) compared with no walking aid. The results confirm our 12 months gait speed trend, in which an increase of 47.2% compared to the pre-operative values was reported [7], [8]. The ActiGait® system shows obviously an improvement of gait speed also in comparison to the orthosis (27.3 s) or the surface stimulation (23.9 s).

Gait endurance increased by 88.12% (202 ± 41 to 380 ± 30 m) over 36 months, and demonstrated that there was even a further increase of the 51.2% improvement, which was already measured in the stroke patient group after 24 months. Furthermore, the risk of fall measured by the Timed-up and go Test was also significantly reduced by 36.6% (from 18.6 to 11.8 s). The recommended placement of the cuff electrode during the surgical procedure with sutures inwards results in a fixed electrode position related to the peroneal nerve fascicles hypothesizing that cuff electrodes will retain this position until final encapsulation and fixation in scar tissue (approximately after 3 weeks post- implantation). Assuming a small statistical inter-patient spread of motor nerve fascicles of the peroneal nerve in the fossa poplitea region as previously investigated by Sunderland [12], we would expect the use of the same channels to trigger dorsalflexion. This assumption was confirmed by the stimulation pattern set during activation with adjacent channels 1 and 4, which were mostly used in our patients (Table 1).

While setting the stimulation frequency parameter, we saw a difference between the stroke patient group and MS patient group. MS patients perceived an uncomfortable electrical stimulation at frequencies above 25 Hz, as compared with 20 Hz and lower. Therefore, during activation we had to find a trade-off between optimal dorsalflexion (higher stimulation frequency) and lack of discomfort (lower stimulation frequencies). This was not the case in stroke patients, where electrical stimulation was not an issue in terms of perceived discomfort.

Based on the questionnaire feedback, 93.9% of the patients (31 of 33) take advantage of the ActiGait® system, which improved their quality of life. The most important factor of this progress was attributed to improved gait safety following reduction of fall risk. Patients are confident to ride public transportations and schedule appointments. This has a positive effect on social interactions and thus on the quality of life and psychological comfort.

## Conclusions

Assessment of long-term results, 36 months post-implantation, proves the ActiGait® system as a very good option for treatment of the centrally induced drop foot. It is reliable in use and demonstrates constantly good results with high patient satisfaction over a long period of time.
