# Supplementary material for: Long-term results following electrical stimulation of the peroneal nerve using the ActiGait® system in 33 patients with central drop foot
Source: Innov Surg Sci. 2021 Jan 8;6(1):3–9. doi: 10.1515/iss-2019-1003 (PMC7790182; doi:10.1515/iss-2019-1003)
Supplement: Supplementary file 1 [file iss-06-20191003-s001.pdf]

Reviewer Assessment

Daniel Martin: Long term results following electrical stimulation of the peroneal nerve using the ActiGait® system in 33 patients with central drop foot

Reviewers’ Comments to Original Submission

Reviewer 1: anonymous

Date received: 07-Jun-2020
Reviewer recommendation: **Return to author for minor modifications**
Reviewer overall scoring: **High**

Assessment Form scores: 5 = High/Yes; 3 = Medium/Adequate; 1 = Low

Is the subject area appropriate for the journal					
Does the title clearly reflect the paper's content?	5				
Does the abstract clearly reflect the paper's content		4			
Do the keywords clearly reflect the paper's content?	5				
Does the introduction present the problem clearly?		4			
Are the results/ conclusions justified?		4			
How comprehensive and up-to-date is the subject matter presented?			3		
How adequate is the data presentation?			3		
Are units and terminology used correctly?	5				
Is the number of cases adequate?	5				
Are the experimental methods/ clinical studies adequate?	5				
Is the length appropriate in relation to the content?	5				
Does the reader get new insights from the article?	5				
Please rate the practical significance.		4			
Please rate the accuracy of methods.		4			
Please rate the statistical evaluation and quality control.		4			
Please rate the appropriateness of the figures and tables.			3		
Please rate the appropriateness of the references.	5				
Please evaluate the writing style and use of language.		4			
Please judge the overall scientific quality of the manuscript.			3		
Are the methods used worthy of reproduction in greater deal?	Yes				
Would you be willing to review a revision of this manuscript?	Yes				

Comments to author: 2-13: As the Actigait implant is not produced anymore, please do not describe it as a new therapy
2-15: ... These findings confirm..: this sentence belongs to results, not objective
4-4: Changes in life quality: please use a standard QOL-Questionnaire.
4-40: SPP not known, SPSS? Why significance $p<0,005$?

4-45: Results: How many of these patients still use the actigait system? You suggest an every day use, is this correct?
Figures 2a, b, c, d: please create professional diagrams

Reviewer 2: anonymous

Date received: 14-Jun-2020
Reviewer recommendation: **Return to author for minor modifications**
Reviewer overall scoring: **High**

Assessment Form scores: 5 = High/Yes; 3 = Medium/Adequate; 1 = Low

Is the subject area appropriate for the journal	5				
Does the title clearly reflect the paper's content?		4			
Does the abstract clearly reflect the paper's content			3		
Do the keywords clearly reflect the paper's content?		4			
Does the introduction present the problem clearly?			3		
Are the results/ conclusions justified?			3		
How comprehensive and up-to-date is the subject matter presented?	5				
How adequate is the data presentation?			3		
Are units and terminology used correctly?			3		
Is the number of cases adequate?	5				
Are the experimental methods/ clinical studies adequate?	5				
Is the length appropriate in relation to the content?		4			
Does the reader get new insights from the article?		4			
Please rate the practical significance.	5				
Please rate the accuracy of methods.			3		
Please rate the statistical evaluation and quality control.					1
Please rate the appropriateness of the figures and tables.			3		
Please rate the appropriateness of the references.			3		
Please evaluate the writing style and use of language.			3		
Please judge the overall scientific quality of the manuscript.		4			
Are the methods used worthy of reproduction in greater deal?	Yes				
Would you be willing to review a revision of this manuscript?	Yes				

Comments to author:
Dear author, thank you for your manuscript and very interesting work. Please see my comments below.

Abstract
LL 13-14: Please adapt the first sentence „Direct electrical stimulation of the peroneal nerve, using the implantable ActiGait® system, enables a new and very efficient therapy of the centrally caused drop foot“

You cant not conclude the efficacy without measuring it. Please clarify in half of a sentence what the AGsystem is.

Introduction

L8: Please replace Routine task by activities of daily living (ADL)
LL13-14: Please correct: damage of the first Moto neuron is not only present in MS and stroke.
L 16: Please replace actual by current treatment.

There is missing a description how the system works and what are the potential benefits in comparison to an orthosis.

Material& Methods.

L 32: how do you define stable MS?
L36: Please repeat Perry's´ definition of drop foot.
L37: Please replace selection by screening
L 39: which kind of electrophysiological testing did you perform?
L46: Please replace computed by assessed
L54: Please replace „recording the distance covered."

Results

Please overall revise the section results and list all the results oft he different tests and different tested scenarios within those test (i.e. no aid vs. orthoses vs. AG at different F-Ups)
When were the other gait tests performed? WHICH SCENARIOS WHERE TESTED WHEN Please enter a figure oft he study design.
L6: which questionnaire did you use?
L22: for which purpose MRI was performed? Why is it that important to identify the bifurcation?
L35: Please correct „while incisions heal" to „had been healed"
Rather explain it more detailed or delete the section „Postoperative care"
Which statistics did you use? Please describe how you tested for significance of the results and which scenarios were analyzed.
Please revise the whole section: The average time required for the 33 patients to cover 20 m during normal gait without walking aids
significantly decreased 36 month following implantation from 31,8±10,2 s to 18,5±4,6 s. Significant walking
speed increase without walking aids was also measured after 6 and 12 month following implantation.
Use of orthesis (27,3±8,3 s) and surface stimulation (23,9±6,5 s) shows the same trend but no significance of
the gait speed increase could be proven.

MRI& Table 1:

Imaging. Can you conclude sth. MRI images-design of the cuff electrode and activation of (mostly) channel 1&4 (Tab.1). Pleas,e analyze again – it would be a very beneficial and intersting

Table 1: what dou think ist he explanation for the different frequencies etc.?

Discussion

The discussion should focus on the dynamic of changes at the longitudinal time course at the three different test time points. Is there a hint for a steady state? Please insert a figure.
L 16: „trainings effect of the cardiopulmonary and musculoskeletal systems" you did not tested for it, please delete.
LL 19,20: Is the TUG test the appropriate test for capturing risk of falling?

Conclusion

Please revise the whole section Conclusion.

Please involve a native speaker to revise grammar and language.

Authors' Response to Reviewer Comments

Date received: 07-Jul-2020

Response to reviewer 1

Thank you very much for your time and effort in reviewing our submitted paper. Please find below our answers:

2-13: As the Actigait implant is not produced anymore, please do not describe it as a new therapy
Done

2-15: ... These findings confirm..: this sentence belongs to results, not objective
Done

4-4: Changes in life quality: please use a standard QOL-Questionnaire.
We used our own simple questionnaire to better express the way Peroneal nerve stimulator influences the Quality of life of the patients. In our opinion a standard Questionair would have not been better. Patient's acceptance to these 4 simple questions was much higher as compared to a more complex, standard questionnaire.

4-40: SPP not known, SPSS? Why significance p<0,005?
Sorry spelling mistake; we used SPSS, indeed.
In choosing the significance value p<0,005 we followed the recommendations of :
Benjamin, D.J., Berger, J.O., Johannesson, M. et al. Redefine statistical significance. Nat Hum Behav 2, 6–10 (2018).
https://doi.org/10.1038/s41562-017-0189-z

4-45: Results: How many of these patients still use the actigait system? You suggest an every day use, is this correct?
All 33 patient use the ActiGait System. Yes it is correct; patients use the system on daily basis.

Figures 2a, b, c, d: please create professional diagrams
I find the diagram expressive enough

Response to reviewer 2

Many Thanks.
Thank you very much for your time and effort in reviewing our submitted paper. Please find below our answers:

Abstract
LL 13-14: Please adapt the first sentence „Direct electrical stimulation of the peroneal nerve, using the implantable ActiGait® system, enables a new and very efficient therapy of the centrally caused drop foot"You cant not conclude the efficacy without measuring it. Please clarify in half of a sentence what the AGsystem is.
Done: Direct electrical stimulation of the peroneal nerve, using the implantable ActiGait® system, enables a therapy of the centrally caused drop foot, improving the gait of the patients.

Introduction
L8: Please replace Routine task by activities of daily living (ADL)
Done

LL13-14: Please correct: damage of the first Moto neuron is not only present in MS and stroke.
Done: This results in a spastic paresis which is hemiparetic in e.g. stroke and monoparetic in Multiple sclerosis (MS) patients

L 16: Please replace actual by current treatment.
Done

There is missing a description how the system works and what are the potential benefits in comparison to an orthosis.
Done: Selective electrical stimulation applied to the fascicles of the peroneal nerve through a 4 channel cuff electrode proximal to the knee joint, contracts the ankle dorsiflexor and everter muscles followed by a balanced dorsiflexion of the foot.

Material& Methods.
L 32: how do you define stable MS?
Done: Less than 3 relapses per year.

L36: Please repeat Perry's´ definition of drop foot.
Criteria for patient selection
Done:

1. *have a hemiparesis persisting for at least 6 months,*
2. *are able to walk 10 m in less than 1 minute with or without a walking aid but without the help of another person*
3. *have a reduced speed of walking*
4. *have a positive response to surface electrical stimulation of the peroneal nerve– i.e. muscle contraction results in ankle dorsiflexion and the ability to achieve normal heel contact*

L37: Please replace selection by screening
Done.

L 39: which kind of electrophysiological testing did you perform?
Done: by means of electrophysiologic testing, measuring Nerve conduction velocity (NCV) and EMG.

L46: Please replace computed by assessed
Done

L54: Please replace „recording the distance covered."
Done: The distance covered by patient while walking for 6 minutes before and after the implantation with and without walking aids (orthosis, surface stimulation, ActiGait® system) was measured

Results
Please overall revise the section results and list all the results oft he different tests and different tested scenarios within those test (i.e. no aid vs. orthoses vs. AG at different F-Ups)When were the other gait tests performed? WHICH SCENARIOS WHERE TESTED WHEN Please enter a figure oft he study design.
In my oppinionare the different Gait tests are sufficiently described

L6: which questionnaire did you use?
We used our own simple questionnaire to better express the way Peroneal nerve stimulator influences the Quality of life of the patients. Patient's acceptance to these 4 simple questions was much higher as compared to a more complex, standard questionnaire

L22: for which purpose MRI was performed? Why is it that important to identify the bifurcation?
One of the manufacturer's recommendations is to avoid inclusion of the sensory branch N. Cutaneus surae lateralis in the cuff electrode to avoid unpleasant sensation/pain due to electrical stimulation. Due to the very limited space between the knee fold and this bifurcation, it is often necessary to further proximally dissect this bifurcation to avoid cuff entrapment (source of most nerve injuries, as experienced worldwide during these implantations. Decision to dissect and the extent of dissection as well as

location or lack of sensory branch (in some cases branching occurs from the tibial nerve) can be performed and planned before surgery based on MR imaging.

L35: Please correct „while incisions heal“ to „had been healed“
Done

Rather explain it more detailed or delete the section „Postoperative care“
Done, added further explanations: The system can be activated 4 weeks after implantation, while incisions had been healed and cuff electrode is fully encapsulated in scar tissue to avoid sliding and rotation around the peroneal nerve. This is to ensure stable and reliable stimulation. During activation all individual channel are tested to determine the foot movement they induce and finally, those that ensure an optimal foot lift (dorsalflexion) and foot balance (eversion or inversion) are selected. Stimulation intensity (pulse width), ramp-up and frequency are also optimized to give a balanced and sufficient foot lift. Follow-ups, to optimally re-adjust stimulation are performed every 3 month during first year and annually later.

Which statistics did you use? Please describe how you tested for significance of the results and which scenarios were analyzed.
Done: We used SPSS software. We tested for the hypothesis that gait parameters Gait speed, Gait endurance and Gait stability using ActiGait are significantly better as compared to same gait parameters without any support

Please revise the whole section: The average time required for the 33 patients to cover 20 m during normal gait without walking aidssignificantly decreased 36 month following implantation from 31,8±10,2 s to 18,5±4,6 s. Significant walkingspeed increase without walking aids was also measured after 6 and 12 month following implantation.Use of orthesis (27,3±8,3 s) and surface stimulation (23,9±6,5 s) shows the same trend but no significance ofthe gait speed increase could be proven.
Done: The average time required for the 33 patients to cover 20 m during normal gait without walking aids decreased significantly measured 36 month following implantation compared to baseline at system activation, from 31,8±10,2 s to 18,5±4,6 s. Significant walking speed increase without walking aids was also measured after 6 and 12 month following implantation. Performing this test using orthesis (27,3±8,3 s) or surface stimulation (23,9±6,5 s) suggests same trend off increased walking speed over time but no significance of the gait speed increase could be proven.

MRI& Table 1:
Imaging. Can you conclude sth. MRI images-design of the cuff electrode and activation of (mostly) channel 1&4 (Tab.1). Pleas,e analyze again – it would be a very beneficial and intersting
Table 1: what dou think ist he explanation for the different frequencies etc.?

The recommended placement of the cuff electrode during the surgical procedure, with sutures inwards, results in a fixed electrode position relative to peroneal nerve fascicles under hypothesis that cuff electrode will retain this position until final encapsulation and fixation in scar tissue (after about 3 weeks following implantation). Assuming a small statistical inter patient spread of motor nerve fascicles within the peroneal nerve in the fossa poplitea region as previously investigated by S. Sunderland [Reference – wird im Paper eingefügt] we would expect the use of same channels to trigger dorsalflexion. This hypothesis was confirmed by the stimulation pattern set during activation with adjacent channels 1 and 4 mostly used in all patients (Table 1). While setting the stimulation frequency parameter, we saw a difference between the stroke patient group and MS patient group. MS patients perceived as uncomfortable electrical stimulation at frequencies above 25 HZ, as compared with 20 Hz and lower. Therefore during activation we had to reach a trade-off between optimal dorsalflexion (higher stimulation frequency) and lack of discomfort (lower stimulation frequencies). This was not the case in stroke patients where electrical stimulation was not an issue in terms of perceived discomfort.

Discussion
L 16: „trainings effect of the cardiopulmonary and musculoskeletal systems“ you did not tested for it, please delete.
Done

LL 19,20: Is the TUG test the appropriate test for capturing risk of falling?
This test assesses gait stability (static and dynamic balance) and thus indirectly risk of falling.

Conclusion
Please revise the whole section Conclusion.
Thank you for comment. Could I ask you to detail the request?

Please involve a native speaker to revise grammar and language.
Sorry for language and grammar, unfortunately I am not a native speaker

Reviewers' Comments to Revised Submission

Reviewer 1: anonymous

No comment povided

Reviewer 2: anonymous

Date received: 23-Jul-2020
Reviewer recommendation: **Accept in present form**
Reviewer overall scoring: **Excellent**

Assessment Form scores: 5 = High/Yes; 3 = Medium/Adequate; 1 = Low

Is the subject area appropriate for the journal		4			
Does the title clearly reflect the paper's content?		4			
Does the abstract clearly reflect the paper's content			3		
Do the keywords clearly reflect the paper's content?		4			
Does the introduction present the problem clearly?			3		
Are the results/ conclusions justified?			3		
How comprehensive and up-to-date is the subject matter presented?		4			
How adequate is the data presentation?			3		
Are units and terminology used correctly?			3		
Is the number of cases adequate?		4			
Are the experimental methods/ clinical studies adequate?			3		
Is the length appropriate in relation to the content?			3		
Does the reader get new insights from the article?		4			
Please rate the practical significance.		4			
Please rate the accuracy of methods.			3		
Please rate the statistical evaluation and quality control.			3		
Please rate the appropriateness of the figures and tables.			3		
Please rate the appropriateness of the references.			3		
Please evaluate the writing style and use of language.			3		
Please judge the overall scientific quality of the manuscript.			3		
Are the methods used worthy of reproduction in greater deal?	Yes				

Comments to author: Thank you for your work and revisions
